# MPOX transmission risks and biosafety protocols in laboratory animal research

**DOI:** 10.1186/s42826-026-00265-x

**Published:** 2026-01-21

**Authors:** Mobolaji Abdulateef Ayoola, Abayomi Oyeyemi Ajagbe, Blessing Simon Oyeleye, Christiana Ololade Olajimbiti, Maryam Ebunoluwa Zakariya, Al-Hassan Soliman Wadan, Ifukibot Levi Usende

**Affiliations:** 1 Department of Veterinary Anatomy, University of Abuja, Abuja, Nigeria; 2 Department of Anatomy, Nile University of Nigeria, Abuja, Nigeria; 3 Department of Veterinary Pathology, University of Abuja, Abuja, Nigeria; 4 Department of Veterinary Public Health, Faculty of Veterinary Medicine, University of Ilorin, Ilorin, Nigeria; 5 Oral Biology Department, Faculty of Dentistry, Galala University, Galala, Egypt

**Keywords:** Monkeypox, Laboratory animal, Biosafety protocol, Biosafety level, Animal research

## Abstract

Mpox, known before now as monkeypox, is a novel zoonotic disease that may infect both humans and animals. It usually spreads by direct contact with bodily fluids, lesion material, or fomites (such as contaminated linens) and prolonged face-to-face contact. Mpox can spread to laboratory animals in a research laboratory through either a general outbreak or during procedures. Animal models are essential as we learn more about how infections occur and how to treat illnesses in both humans and animals which require laboratory procedures with mpox virus. As drug-resistant organisms proliferate, bioterrorism becomes a greater danger, international trade and travel expand, and the number of newly developing infectious illnesses grows, the use of animals in infectious disease research has increased. Animal research offers a solid basis for clinical trials used in standard medication development. Animal studies must be planned to produce conclusive effectiveness evidence that is robust, rigorous, and repeatable per the animal rule so that the agency may depend on the results when making regulatory decisions. There is a need to understand the transmission risk and biosafety protocol to be in place in laboratory settings to prevent an outbreak and probably contain an outbreak. Hence, this review discussed the overview of mpox in animal research, biosafety protocols in mpox research, laboratory waste management methods, legal and ethical considerations in mpox research, challenges in carrying out mpox research and future directions.

## Background

**Fig. 1:**
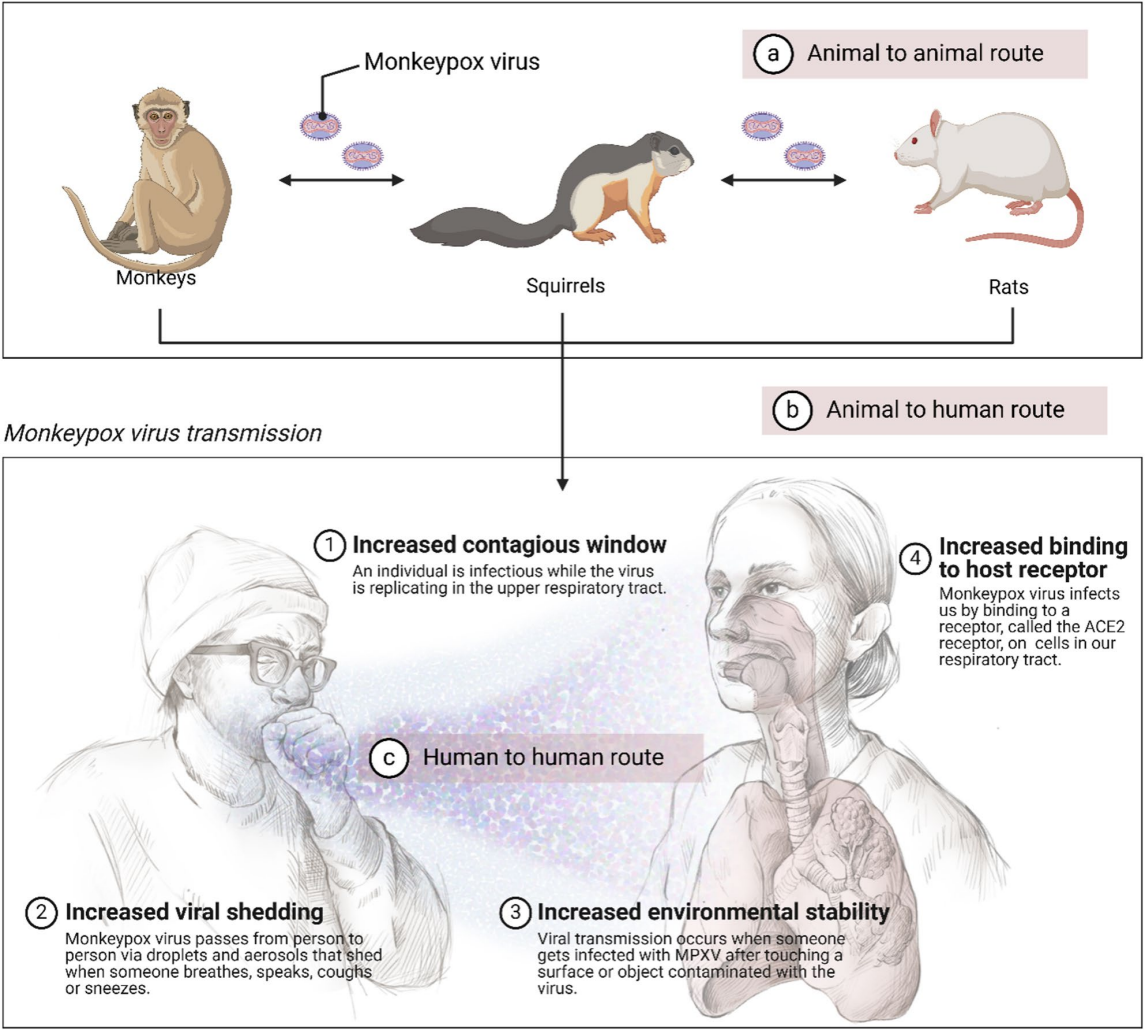
Monkeypox virus transmission pathways and factors. There are three routes of mpox transmission which are (**a**) animal to animal route which involves the spread of mpox among laboratory animals including rats, squirrel and monkeys, (**b**) animal to human route which occur during contact with infected laboratory animals or exposure to aerosol in the laboratory and (**c**) the human-to-human route which can be through contact or aerosol from infected laboratory personnel to another. Additionally, the figure shows the factors responsible for the mpox which are (1) contagious window (2) virus shedding (3) environmental stability (4) binding to host receptor

An infection with the mpox virus (MPXV) causes mpox. Known before now as monkeypox, mpox is a novel zoonotic disease that may infect both humans and animals [1–3]. Direct contact with bodily fluids, lesion material, or fomites (such as contaminated linens) and prolonged face-to-face contact are the main ways that the mpox virus, a zoonosis, can spread from person to person [4]. Since it is an orthopoxvirus, respiratory droplets are the main way that it spreads [4]. The disease was discovered to exist in a variety of species, such as rats, squirrels, and other small mammals, as well as in people in central and western African nations after it was initially discovered in 1958 in monkeys housed for scientific purposes in Denmark (Fig. 1) [2, 3, 5].

Rapid globalization, population migration, and expanding trade networks have all played a part in the international spread of mpox, which has led to outbreaks in a number of nations throughout the world [1]. Notably, 110 nations and areas were impacted by a global Mpox outbreak in 2022 [1, 5, 6]. Even though the World Health Organization stated in May 2023 that mpox outbreaks were no longer considered “a Public Health Emergency of International Concern,” it is crucial to note that the virus’s quick evolution and increased international travel have led to an increase in mpox cases in some Asian regions [1]. In this review, we have focused to discussed the overview of mpox in animal research, biosafety protocols in mpox research, laboratory waste management methods, legal and ethical considerations in mpox research, challenges in carrying out Mpox research and proposed future directions.

### The need for animal research to understand the disease and develop countermeasures

Animal models are essential as we learn more about how infections occur and how to treat illnesses in both humans and animals [7]. As drug-resistant organisms proliferate, bioterrorism becomes a greater danger, international trade and travel expand, and the number of newly developing infectious illnesses grows, the use of animals in infectious disease research has increased [7]. Animal research offers a solid basis for clinical trials used in standard medication development. Animal studies must be planned to produce conclusive effectiveness evidence that is robust, rigorous, and repeatable per the Animal Rule so that the agency may depend on the results when making regulatory decisions [8].

**Fig. 2:**
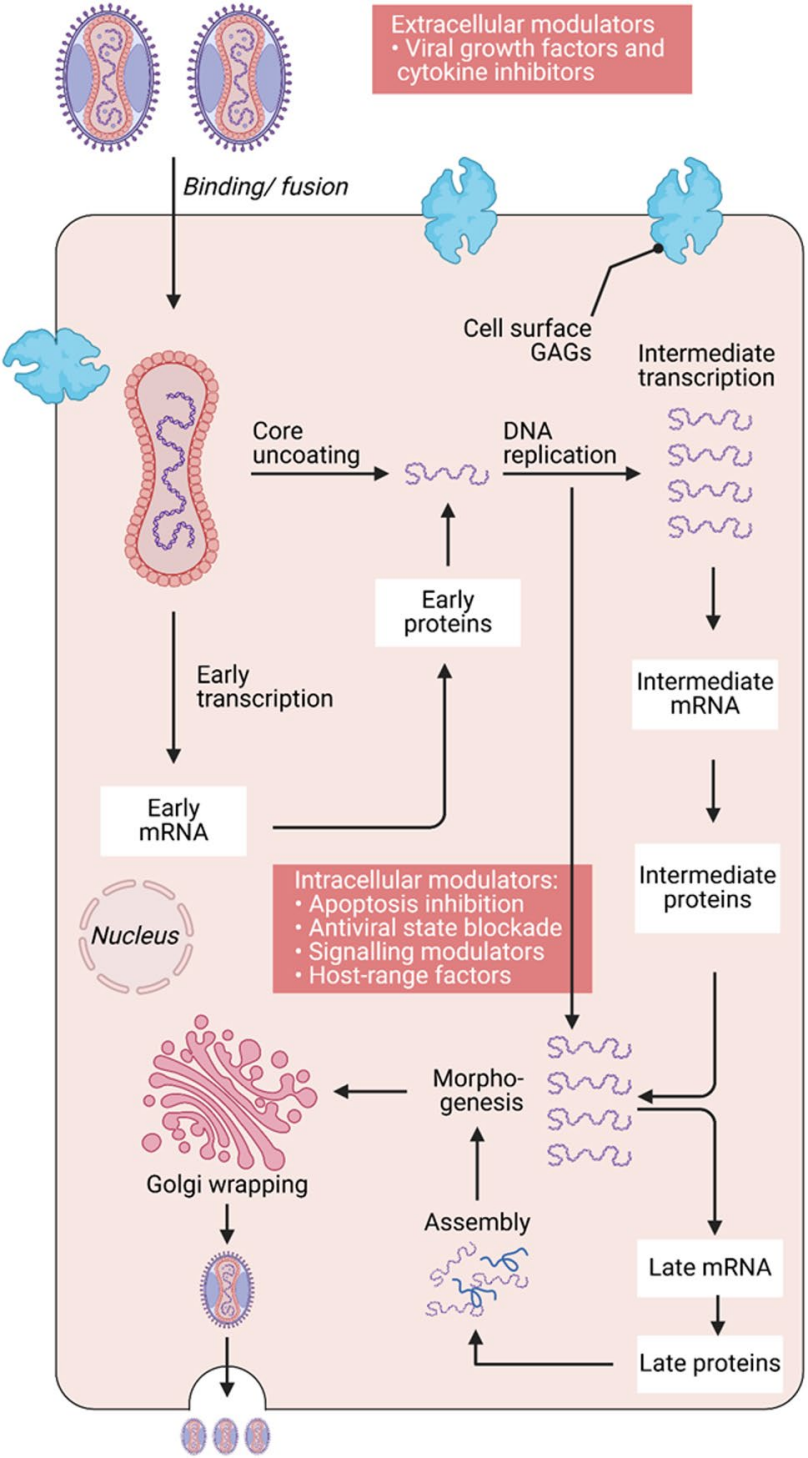
Molecular mechanism of mpox viral replication. The molecular mechanism of mpox viral replication, there are ten steps which are viral binding and fusion, core uncoating, early transcription/ translation, deoxyribonucleic acid (DNA) replication, intermediate transcription/translation, Late transcription/translation, assembly, morphogenesis, golgi wrapping and the release of the mature virions

There are currently no antiviral medications or vaccinations that are specific to MPXV. Researching pharmaceutical countermeasures, including a detailed account of the pathophysiology and lesions in susceptible hosts, requires the development of MPXV animal models. The majority of animal models used to study the characterization are generated from historical isolates of MPXV [2]. Therefore, more research is required to determine the pathophysiology of circulating strains of MPXV which we have summarized in Fig. 2.

### Importance of safety protocols in mpox research involving animals

Direct contact with skin wounds, scabs, rashes, saliva, respiratory secretions, and bodily fluids through ingestion of contaminated tissues and animal bites is the primary way that MPX is spread from infected animals to human [9]. Transmission from humans to laboratory animals is a serious event that could potentially lead to the virus establishment in wildlife and the disease evolving into an endemic zoonosis, which would further spread the Mpox virus among the laboratory attendance and general population [4]. This is because rodents and specific species of the Sciuridae family (squirrels) are likely to be more suitable hosts than humans [4, 10].

Thus, precautions including wearing personal protective equipment (PPE), maintaining enough ventilation, and adhering to the biosafety level (BSL) are essential to avoiding unintentional infections that can cause more serious public health issues [4].

Conclusively, safety procedures serve as an essential safeguard against accidental spread to people or other animals, making sure that research doesn’t unintentionally fuel new outbreaks. This is especially crucial in light of the increasing awareness of zoonotic illnesses and their consequences around the world.

## Overview of Mpox in animal research context

### Natural hosts and species susceptible to mpox

**Fig. 3:**
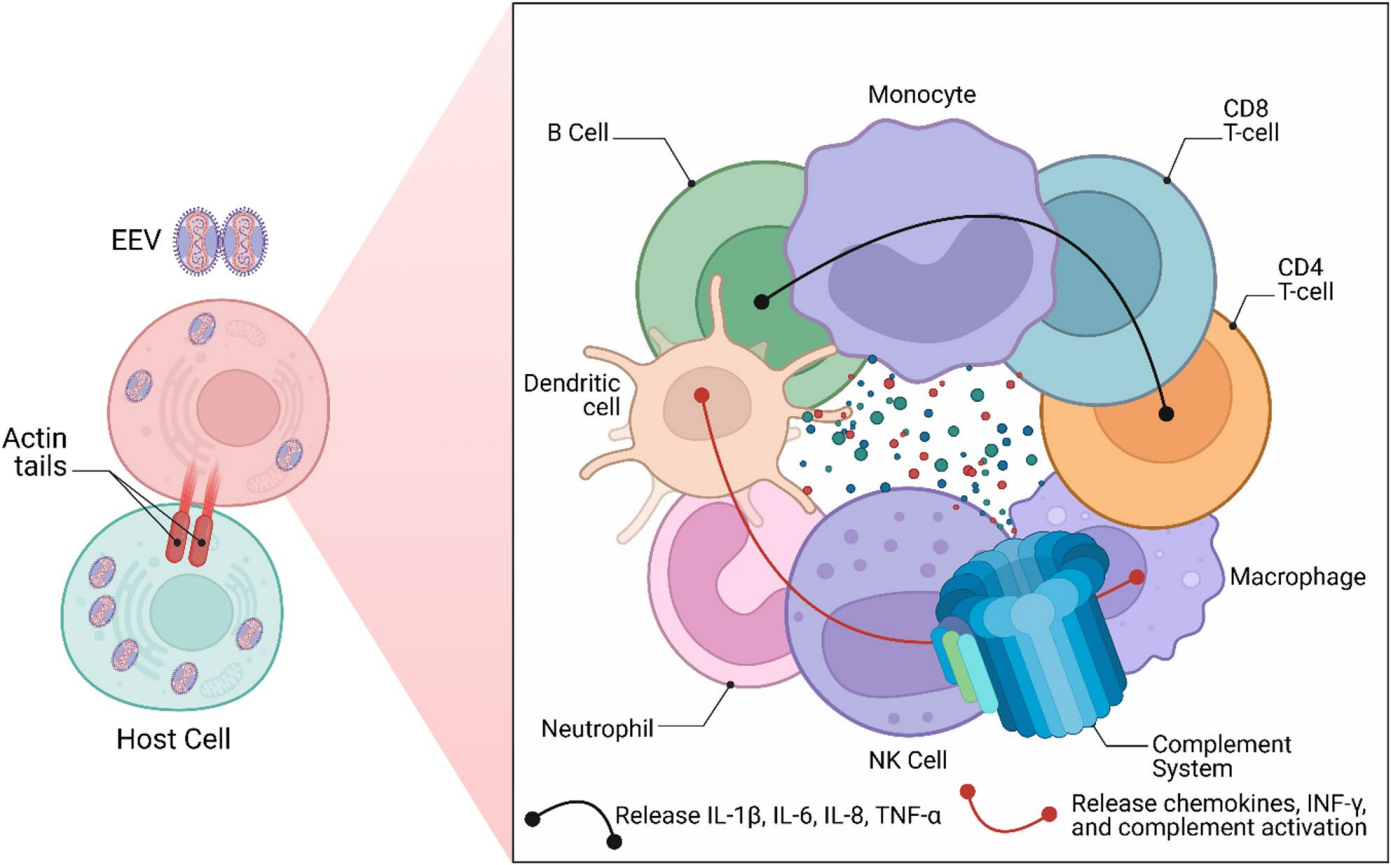
The immune response of the host cell to mpox virus infection involves both specific and non-specific mechanisms. Upon entry of the Mpox virus into host cells, mononuclear phagocytes and neutrophils are activated, promoting recruitment and enhanced infiltration of immune cells. Simultaneously, antigen-presenting cells such as dendritic cells are stimulated, triggering the release of effector molecules and chemokines. Other components of the immune system, including T cells, B cells, natural killer (NK) cells, and the complement system, also activate their respective effector functions. Key players in this immune response include interleukins (IL), helper T cells (Th), interferons (IFN), and antibody-dependent cell-mediated cytotoxicity (ADCC)

MPXV has been isolated from *Funisciurus anerythrus* and *Cercocebusatys*, and it has been shown that several wild animal species, including rodents and nonhuman primates (NHPs), are vulnerable to the virus; however, the natural reservoirs of MPXV are yet to be established [2, 6, 9]. Mpox’s precise animal reservoir is unknown, although rodents—such as giant-pouched rats, African dormice, and squirrels—are known to carry the virus as hosts and are thought to keep it alive in the environment via immune response of the host cell to mpox virus infection which involves both specific and non-specific mechanisms (Fig. 3) [6]. In addition to nosocomial and home transmission, humans can get MPXV by direct contact with sick animals or contaminated objects, animal bites or scratches, or consumption of meat from infected animals [2, 9].

### Common animals used in mpox research (e.g., non-human primates, rodents), rationale for studying mpox in these animals and potential risks

Animal models are essential for assessing the effectiveness of antiviral medications and MPXV vaccines. Table 1 shows the common animals models used for mpox resaerch, rationale and the potential risk associated with its use. Although they are too costly to be widely employed, nonhuman primates who are infected with MPXV exhibit pathophysiology and symptoms that are most comparable to those of humans [11]. Models of rodents are less expensive, simpler, and less hazardous to handle. To develop mpox countermeasures, the highly vulnerable CAST/EiJ mice model has been extensively utilized [11].

**Table 1:** Animal models for mpox research

s/*n*	Animal models utilized	Rationale	Potential risk	References
1	Mice of different types, namely CAST/EiJ mice, BALB/c mice	Low mortality, economic accessibility, genetic uniformity, a robust immune system, the capacity for rapid reproduction, and a wide range of immunological reagents.	Absence of clinical symptoms as observed in humans. The median lethal dosage (LD50) of 10^7^ was high because no MPXV-infected animals experienced mortality.	[2, 11]
2	Guinea pigs	Availability of immunological reagents	The infected animals did not exhibit any clinical symptoms.	[2, 12, 13]
3	Rat species, namely white rats, multimammate rats, and cotton rats	The readily available inbred cotton rats and relevant commercial chemicals	The West Africa (WA) strain of MPXV did not cause any clinical signs in adult white rats, whether it was injected, intranasally, or topically applied.	[2]
4	Hamster	Availability of immunological reagents	Absence of clinical symptoms	[2, 12]
5	Ground squirrels (African rope squirrels)	Susceptible to MPXV infection spontaneously.	MPXV infection can cause serious illness in African rope squirrels that shed a lot of viruses, as evidenced by the fact that an animal kept in the same room but in a different cage also exhibited severe clinical disease of the virus.	[2]

## Biosafety in animal research facilities

### Biosafety levels (BSL) and animal biosafety levels (ABSL) requirements

The ABSL are a framework that provides containment mechanisms to safeguard personnel, animals, and the environment from dangers when working with animals in laboratories [14]. Vaccinated staff are advised to process and test mpox suspected specimens in biosafety level 2 (BSL-2) facilities [4]. If however the staff is not vaccinated, the usage of a BSL-3 facility is recommended [15]. A BSL-2 containment facility and procedures could be used for analysis on non-lesion specimens (e.g., urine, blood), culture/sensitivity testing, and DNA extraction. Laboratory waste should be decontaminated on-site before disposal [15].

### Differences between biosafety levels (BSL) 2 and 3, and higher containment protocols

There are four animal biosafety levels.

#### ABSL 1

used for animal work with agents that have been shown not to infect normal human populations [16].

#### ABSL 2

for animal experiments with moderately risky agents that mainly affect human through the mucosal or oral route. Class I, II, or III biosafety cabinets are required [14].

#### ABSL 3

used in animal work with agents that can cause fatal disease in humans and may be airborne [15].

#### ABSL 4

used for animal-related research where the agent is non-exotic and potentially lethal human diseases for which there is no cure or vaccine. Class III biosafety cabinets are required (Cena-Navarro, 2024).

### Biosafety protocols in mpox research

**Fig. 4:**
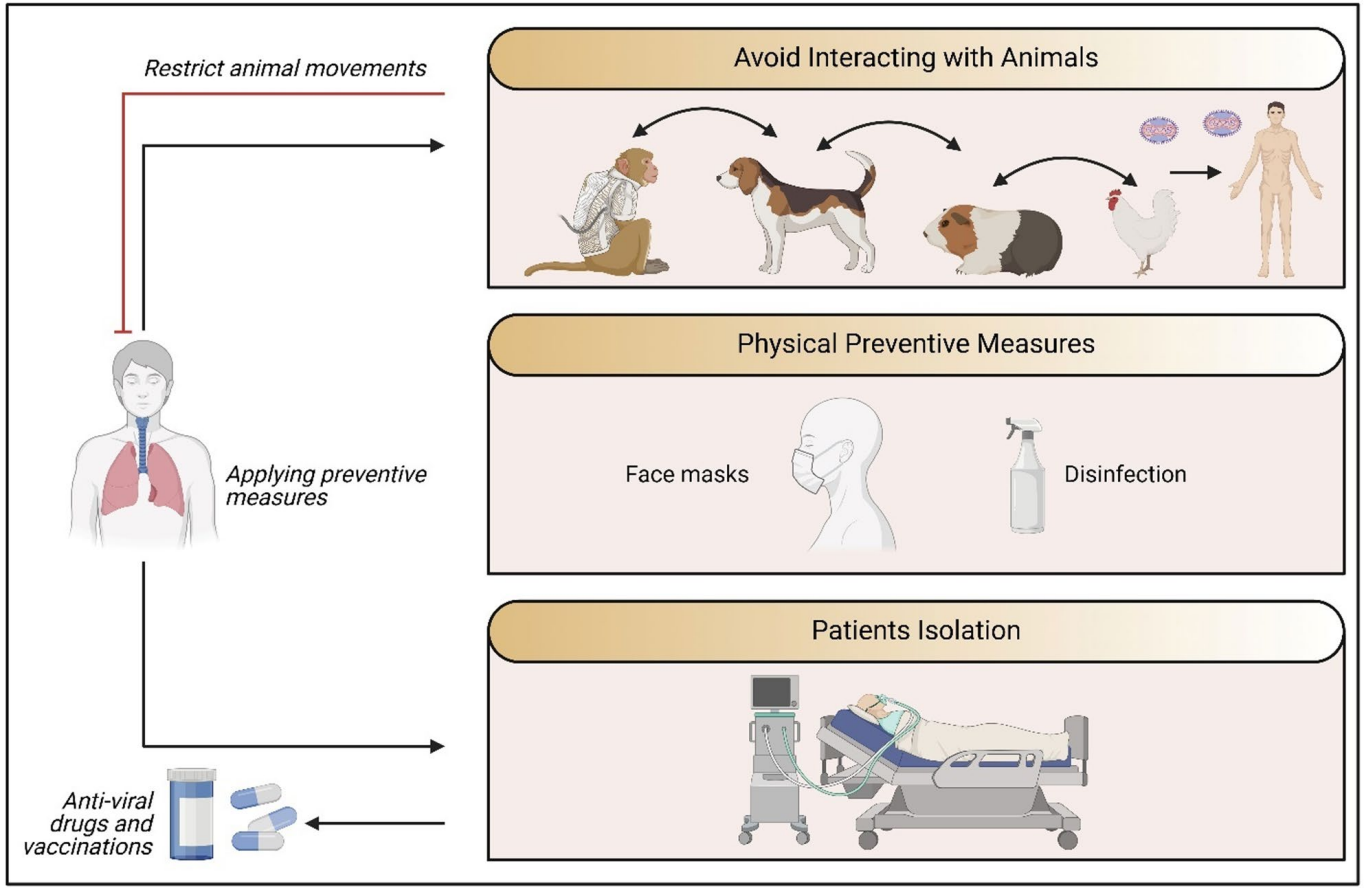
Biosafety protocols in mpox research. Outlined ways to prevent Mpox, including limiting animal movement, preventing animal contact, use of face masks, disinfection, patient isolation, and antiviral medications or vaccines. In order to prevent and manage the spread of zoonotic diseases, these tactics combine to establish a One Health approach that addresses the health of humans, animals, and the environment

The biosafety protocol for mpox research is outlined in Fig. 4 and includes avoiding unprotected interaction with laboratory animals, using protective equipment, and vaccination. In the next section we focused on disinfection and vaccination.

#### Disinfection

Studies have shown that the mpox virus can be effectively inactivated by ≥ 4 log₁₀ reduction using 70% ethanol with a 1-minute contact time or 0.2% peracetic acid with a 10-minute exposure, both in suspension assays and on contaminated surfaces [17]. According to Kampf [17] suspension assays, 14.4% hydrogen peroxide and iodine concentrations from 0.04% to 1% demonstrated effective virucidal activity against mpox virus. Sodium hypochlorite (0.25–2.5%, 1-minute contact time), 2% glutaraldehyde (10 min), and 0.55% orthophthalaldehyde (5 min) were effective disinfectants on experimentally contaminated surfaces [17]. The United Kingdom Health Security Agency (UKHSA) has documented products such as NeuMoDx vantage viral lysis buffer (50.5% guanidine hydrochloride, 0.8% sodium tetraborate decahydrate, and 0.3% Tris(2-carboxyethyl) phosphine hydrochloride), Zymo research DNA/RNA shield buffer (SafeCollect Swab Tube), and Qiagen buffer ATL (which includes sodium dodecyl sulfate at concentrations ≥ 1 < 10% w/w) to be efficient in viral inactivation [18]. Roche cobas PCR media (with ≤ 40% guanidine hydrochloride), Longhorn vaccines and diagnostics primestore molecular transport medium (20–30% guanidine thiocyanate, 19–25% ethanol, and < 0.7% N-Lauroylsarcosine Na+), and Qiagen buffer AVL (50–70% guanidinium thiocyanate) [18]. Furthermore, 70% ethanol, thermo scientific inhibiSURE viral inactivation medium, severn biotech Ltd L6 buffer, BioServ MagBead viral RNA lysis buffer (1–2% dl-dithiothreitol and 20–30% guanidine thiocyanate), hologic panther fusion specimen lysis tubes, and E and O laboratories Ltd molecular sample solution (with 40–50% guanidine thiocyanate and 0.5–1% Tergitol 15-S-9 surfactant) were also effective [18, 19].

#### Vaccination

The Advisory Committee on Immunization Practices (ACIP) has recommended that personnel with potential exposure to the mpox virus should take pre-exposure orthopoxviral vaccination [20]. JYNNEOS and ACAM2000 are the two vaccines authorized for the prevention of mpox [20]. Full immunization is achieved four days after the administration of the second dose of JYNNEOS, or four weeks after a single dose of ACAM2000 [19, 20]. Based on these guidelines, the biosafety in Microbiological and Biomedical Laboratories (MBL) also advised vaccination for laboratory personnel who handle cultures of Monkeypox virus [19]. The BMBL and ACIP prescribed that individuals with Monkeypox virus receive JYNNEOS booster vaccinations every two years or ACAM2000 boosters every three years [19, 20].

### Facility design and containment

#### Air filtration systems, secure enclosures, waste disposal procedures

Air filtration is the most common and frequently used easy and effective approach for capturing aerosols in indoor air environments [21]. It uses materials such as air as fiberglass, carbon, or polymers as filters. There are two categories of filters based on high efficiency; they include high-efficiency particulate air (HEPA) and ultralow particulate air (ULPA) filters [22]. Facility designs should have a general plan, which usually includes a multilevel concentric layer of increased physical movement from an outer perimeter to the inner containment. The innermost part of the facility should be secured and exclusive. These areas are where there are high containment laboratories and computer network hubs with data management and bioinformatics resources [23].

### Risk assessment for researchers and laboratory personnel

Risk assessment is conducted mainly to limit or decrease potential harm. It includes carrying out techniques such as biohazard containment, hazard elimination or control, occupational health programs, and institutional committee oversight. It would help to reduce risks and increase safety. In low-income countries with limited resources, the necessity of risk management in animal research facilities cannot be overemphasized. Implementing realistic and cost-effective measures to prevent disease spread and protect human and animal health is crucial in achieving far-reaching benefits for both local communities and the worldwide research community [14].

### Occupational health and safety

#### Potential risks to researchers handling mpox-infected animals

The use of laboratory animals for research presents varying risks to humans, animal handlers, researchers, and the environment. People in the community and other animals may also be at risk. The need for an in-depth risk assessment to help in formulating mitigation controls is therefore of great importance. In working with laboratory animals during research, potential hazards could include physical, zoonotic diseases, sharps, allergens, vectors, escape, or biosecurity [14]. Animals’ claws and teeth could pose danger of injuries to handlers during manipulation. Another risk could be the escape of animals. Infected animal escapees could be a potential risk of other animals in the laboratory, laboratory workers and the environment being contaminated/infected from the laboratory. Considerations to ensure safety must be made while maximizing available resources [14]. The chance of occupational vulnerability is low for personnel pursuing proper biosafety approaches. Unprotected exposure carries a high likelihood of infection and a moderate risk of the disease (due to the direct exposure of mucosae to a potentially substantial quantity of virus). The danger for unprotected laboratory staff is estimated to be high. Laboratory managers, personnel, and occupational health officers for each facility must perform site- and activity-specific risk assessments for mpox testing to determine whether enhanced safety precautions are needed [24].

#### Strategies to minimize researcher exposure (e.g., personal protective equipment (PPE), training)

It is crucial to minimize the risk of laboratory transmission as much as possible; hence, laboratories conducting mpox research should take appropriate steps. These steps could include the use of appropriate PPE, limiting access to samples and ensuring only trained professionals process the samples or handle the animals infected with mpox [4]. For serological assays and non-transmissible diagnostic tests, a BSL-2 laboratory and the use of a class II biological safety cabinet (BSC) are recommended. In cases of laboratory procedures that involve dealing with a live virus, such as virus culture, a BSL-3 is needed [25]. Measures should be taken when testing clinical samples from suspected or confirmed animals. Mitigation controls to prevent the escape of animals should be considered in the laboratory design and standard operating procedures. Laboratory workers should work in synergy with the animal facility manager, biosafety officer, and other stakeholders to ensure safe and secure implementation of their research [14]. To mitigate the potential of aerosol production, pipetting may be performed using filter tips. Pipette contents may be expelled close to the targeted surface or by allowing the fluid to flow down the inside of the container. Centrifuge buckets should be opened inside the BSC. If a BSC is not available, splash barrier protections such as a bench or instrument shield may be used [16, 26, 27].

Biosafety consists of a mix of primary and secondary barriers between laboratory personnel and the biologic agent, facility practices, and additional safety devices such as PPE. Physical safety equipment, such as biological safety cabinets (BSCs), provides immediate protection against hazards. Additional primary containment devices include enclosed containers, centrifuge safety cups, and sealed containers [16]. Facility design can provide a supplementary barrier to dangers by including appropriate ventilation, anterooms, and airlocks. PPE is generally considered a secondary barrier. PPE use varies based on laboratory tasks and specimens. PPE typically includes lab coats, eye and facial protection, and solid-front gowns with cued sleeves and gloves [27]. Laboratories that process and test mpox lesion materials, including swabs of lesion surface and exudate, and lesion crusts, should have the necessary equipment, engineering controls, PPE, appropriate diagnostic assays, and properly trained personnel.

#### Regular health monitoring and vaccination (e.g., smallpox vaccine) for researchers

As with all procedures, laboratories should perform a site-specific and activity-specific risk assessment to identify and mitigate risks. Risk assessments and mitigation measures depend on the procedures performed, hazards involved in the processes and procedures, the competency level of the personnel who perform the procedures, the laboratory equipment and facility, the resources available, and the vaccination status of the personnel who perform the procedures.

For assessed high biosecurity risks, personnel should be comprehensively screened, either uniformly or proportionately according to their roles and accessibility to high-consequence material and the facilities that contain them. Initial personnel screening should be done before employment; regular checks and monitoring should also be carried out frequently.

## Decontamination and waste management

### Decontamination

Procedures like cleaning, disinfection, or sterilization can be employed, depending on the degree of decontamination that must be accomplished. The application will determine which decontamination method—chemical or physical—is used. For example, steam exposure or chemical treatment may be used to decontaminate waste, surfaces, medical devices, or specimens [28]. To choose the best decontamination technique, one must weigh the benefits and drawbacks of the various approaches.

#### Cleaning

Generally speaking, cleaning is the process of getting rid of anything that isn’t a part of the item. Cleaning serves two purposes in laboratory biosafety: (i) it can eliminate organic matter and grime from an object that could deactivate chemical disinfectants or prevent them from coming into contact with biological agents in the object [29] and (ii) it can eliminate a significant percentage of biological agents, increasing the efficacy of following chemical disinfection in reducing them to acceptable levels. Cleaning alone is not a sufficient method of disinfection. It is subjective to determine if an object needs to be cleaned before chemical disinfection; if it appears physically clean, washing is not necessary [30].

#### Disinfection

The existence or lack of a cell wall skeleton, lipid membranes, layers of polysaccharides, and peptidoglycan will determine how resistant or sensitive biological agents are to chemical compounds [31]. Spores are typically more difficult to inactivate than encapsulated viruses, requiring larger quantities of highly active disinfectants. For general surface disinfection, a sodium hypochlorite solution with 1000 ppm (parts per million) of available chlorine is usually adequate; however, stronger solutions (such as 5000 ppm or 10,000 ppm) are advised when handling severe contamination, the presence of organic matter, or biological agents that are resistant to disinfectants.

The risk assessment concludes that gaseous disinfectants, often referred to as fumigants, are necessary in a few specific circumstances, primarily in laboratories with maximal containment measures, in order to sanitise the laboratory area, furnishings, and/or equipment. Gaseous disinfectants may be required when the laboratory has extensive contamination in hard-to-reach places or when equipment needs to be removed from a contaminated area or cleaned before maintenance [32]. Fumigation with formaldehyde gas produced by heating paraformaldehyde or boiling formalin solutions can be used to disinfect rooms and equipment.

The most popular physical technique for decontaminating biological substances is heat. It is possible to employ both moist and dry heat [33]. For steam sterilisation to work, the biological agents must come into contact with water; in other words, the steam must reach every surface or item that has to be cleaned. The best results are obtained while autoclaving with moist heat. The gold standard for disinfecting solid wastes is autoclaving previously cleaned goods in a well-maintained autoclave with interlocked doors and a validated programmable cycle [34].

#### Sterilization

Sterilization is used when a complete elimination of any biological agent, including spores and prions, is necessary; for example, for medical items and waste if the risk assessment indicates the need for very strict decontamination procedures. Sterilization can be achieved using several decontamination methods, such as autoclaving, certain chemical disinfectants, gaseous disinfection combined with a strict SOP, and irradiation [35].

### Waste management

In addition to the risk of environmental contamination, improper handling of laboratory waste raises the possibility of infection, negative consequences, and harm to patients, waste handlers, medical personnel, and the general public. All medical waste products must be segregated, managed carefully, and disposed of safely at the time of creation [36].

#### Minimization and recycling

Source reduction, recycling, residuals disposal, and treatment are all part of the waste management hierarchy [37]. The first two elements that could reduce the quantity of laboratory waste that needs to be disposed of are basically used in waste reduction, which is related to waste management. Waste minimisation benefits both the environment that generates waste for disposal and the environment that receives garbage. Liabilities associated with the disposal of medical waste are also reduced, as are the costs of procuring goods and treating trash [38].

#### Reduction at source

Some forms of reduction entail actions that either entirely stop the usage of a material or produce less trash [39]. Examples include switching from mercury thermometers to digital electronic thermometers, working with suppliers to reduce product packaging, and switching from toxic chemical cleaners to non-toxic biodegradable cleaners.

#### Recycling

Recycling is the process of gathering trash and turning it into something new [40]. In the research laboratory, a lot of things can be recycled. Recycling is simple for materials like organics, plastics, paper, glass, and metal.

#### Re-use

Reusing something is using it repeatedly for the purpose for which it was intended, not trying to find a new application for it [41]. To promote recycling, choose for reusable things whenever feasible rather than throwaway ones. Another prerequisite for reuse is establishing reliable standards for the sterilization and cleaning of tools and materials that can be used again.

#### Color coding system

By using a color-coding scheme, it is possible to quickly and clearly identify the risks related to the particular type of laboratory waste being handled or treated. In that regard, the color-coding scheme ought to be kept basic and utilized consistently across the nation. Table 2 below is a table of the color coding system.

**Table 2:** Colour coding of waste bins [42]

Colour of container/bag	Type of waste
Black	Non-infectious dry waste
Green	Non-infectious wet waste (kitchen waste, etc.)
Yellow	Infectious and pathological waste
Yellow with Black band	Chemical waste, including heavy metals
Orange	Radioactive waste
Red	Sharps and Pressurized containers

### Waste collection and transportation

One of the most important aspects of managing laboratory waste is proper collection and transportation. All laboratory professionals must collaborate, and the institution’s housekeeping and maintenance staff must be intimately involved in its execution [43]. Waste collection procedures should be designed to reduce worker risks while ensuring an effective waste flow from the sites of creation to storage or treatment.

#### On-site collection

The site of production should not see a buildup of waste. When trash bags are approximately three-quarters full, the laboratory assistant and other staff members should ensure that they are tightly closed or sealed. Heavy gauge bags definitely need plastic sealing tags of the self-locking variety, although light gauge bags can be closed by tying the neck. Staples shouldn’t be used to close bags. Sharps containers that have been sealed up should not be disposed of in a labeled, yellow infectious health care waste bag [44].

#### On-site transport

Wheeled trolleys, containers, or carts intended only for the purpose of moving rubbish about the establishment may be used. The following requirements as stated by Pasupathi et al. [45], should be met by the on-site transportation vehicle. It should be easy to load and unload; there should be no sharp edge that could damage waste bags or containers during loading and unloading and easy to clean.

#### Off-site transportation of health care waste

The packing and appropriate labeling of trash that will be transferred off-site for treatment and disposal are the responsibility of the laboratory waste generator. Packaging and labeling must be in accordance with national regulations governing the transport of hazardous waste while also stating that there is no risk involved in ensuring that the waste is appropriately handled and disposed of in a disposal facility that has been granted approval [46]. Tracking of waste could be done with the implementation of a consignment system.

##### Consignment notes

Only an accredited transporter or carrier may transfer any laboratory waste to an authorized off-site waste treatment facility. All medical supplies removed from the laboratory facility for treatment or disposal must be recorded on a consignment note that has been properly filled out by the authorized transporter [47]. A copy of the consignment note must be kept on separate records by the generator and the transporter.

### Waste treatment

The goal of treating medical waste is to alter its biological and chemical makeup in order to reduce its potential for harm [48]. The level of treatment is indicated by a number of phrases, including decontamination, sterilization, disinfection, rendering harmless, and killing. These definitions don’t offer a way to gauge how efficient a procedure is. In order to accurately describe the level of microbial annihilation achieved by any health care waste treatment method, words and criteria must be specified. Disinfection is classified as low, intermediate, or high depending on the survival probability of particular microbial groups, while sterilization is defined as a 6log10 survival probability of the most resistant microbe of interest in a given process [49]. However, a growing international consensus for defining levels of microbial inactivation for laboratory waste disinfection is as follows:

Level I: inactivation of vegetative bacteria, fungi, and lipophilic viruses at a 6 log10 reduction or greater.

Level II: inactivation of vegetative bacteria, fungi, lipophilic/hydrophilic viruses, parasites, and mycobacteria at a 6 log10 reduction or greater.

Level III: inactivation of vegetative bacteria, fungi, lipophilic/hydrophilic viruses, parasites, and mycobacteria at a 6 log10 reduction or greater; and inactivation of *B. stearothermophilus* spores and *B. subtilis* spores at a 4 log10 reduction or greater.

Level IV: Mycobacteria, parasites, hydrophilic and lipophilic viruses, fungi, vegetative bacteria, and *B. stearothermophilus* spores are all inactivated at a decrease of at least 6log10.

## Ethical and legal considerations in animal research safety

### Ethics of using animals in mpox research

Natural and experimental monkeypox virus (MPXV) infections in animals have produced important insights into the routes of transmission, pathogenicity, disease ecology, conservation effects on wildlife, and control strategies such as vaccination [50]. Many animal models have been employed to draw insights on this disease [2].

Over the years, various debates have sprung on whether it’s appropriate to use animals for medical research due to findings of their sentential nature; this represents a change from historical viewpoints that considered animals as painless beings [51]. This led to the creation of broad laws or rules that penalize mistreating animals in many nations, but only a few of them specifically address the use of animals for scientific research. This gap is filled by the Council for International Organizations of Medical Sciences (CIOMS), which offers a global ethical framework for the use of animals in biomedical research through its International Guiding Principles for Biomedical Research Involving Animals [52].

In addition, the issue of animal ethics in research has been examined in academic contexts for years, and it remains a contentious social topic, influenced by factors such as housing conditions, the perceived diminishing necessity for such experiments due to the rise of alternative methods, and the ethical concerns surrounding the pain and potential death of animals involved [53].

Animal models have been important in biomedical research for several decades. They are essential for translational research, including MPXV, as they offer invaluable insight into the aetiology of diseases, therapeutic and preventive targets, and treatment approaches [54]. However, there are several ethical and scientific issues surrounding the use of animal models in research, but the general agreement is that only animal studies that have predictable advantages that justify the risks to the animals should be permitted [55]. Because of this, many countries have stringent regulations governing animal research, and every study using animals must first pass an ethical review [56]. Also, many of these ethical reviews are not uniform, and rather than aiming for harmonization as the ultimate objective, effort should focus on improving ethics evaluation for animal research [57]. Therefore, it is crucial to provide these animals with the greatest care possible from an ethical and scientific standpoint, as subpar treatment can cause pain to the animal and compromise the research results [51].

Millions of animals are utilized annually for research, enduring discomfort before being sacrificed [58]. Russell and Burch of the Universities Federation for Animal Welfare (UFAW) released the Principles of Humane Experimental Technique in 1959, outlining the Three Rs—replacement, reduction, and refining— [59]. By creating substitute techniques, technological advancement has helped in solving the issues surrounding animal research [60]. It includes non-testing methods such as expert systems and testing methods such as in vitro, in vivo, in silico, ex vivo, or in chemico reduced/refined procedures [58]. However, even with the growing knowledge of these principles, there is still room to improve their application.

### Legal and institutional regulations

The use of animals in experiments has changed for a number of reasons, and MPXV is not an exception. These include the need to prove the validity of animal research, the diversification of animal experiments, the enhancement of the legal framework concerning experimental animals and animal research, and the actions of animal welfare organizations [61]. In some countries, policies such as institutional animal care and use committees (IACUC) have been created to supervise the care and utilization of research animals and enhance the caliber of scientific work carried out at institutions [62].

In the United States, Institutional Animal Care and Use Committees (IACUCs) under the Public Health Service Policy and the Animal Welfare Act, are required to ensure the ethical and humane treatment of animals used for research in institutions [63]. As a key guideline for carrying out their animal care and use programs, these institutions must adhere to the Guide for the Care and Use of Laboratory Animals [62].

Lack of proper training, irrational expectations for researchers or instructors, cultural misinterpretations, insufficient study monitoring, or an institutional culture that does not encourage compliance can all lead to noncompliance of these regulatory standards. The IACUC has the power to investigate claims of noncompliance and, if required, halt ongoing animal research [64].

MPXV is a highly contagious zoonotic disease caused by a virus [65] and thus requires adherence to regulations. In the US, MPXV is one of the select agents and toxins that is regulated by the Federal Select Agent Program because of the threat they pose to people, animals, or plant health [66]. Also, the implementation of EU Directives 2000/54/EC on the protection of workers against hazards associated with exposure to biological agents at work is mandatory for Member States of the European Union.

The World Health Organization (WHO) Laboratory Biosafety Manual has been widely used at all levels of clinical and public health laboratories and other biomedical sectors as a de facto global standard that outlines best practices and establishes trends in biosafety [67]. Also, the Food and Agriculture Organization, World Organization for Animal Health, and WHO collaborated to create the Tripartite Zoonoses Guide, which offers guiding principles, best practices, and solutions to help countries address zoonoses through a multisectoral, One Health approach [36].

### Training and preparedness for laboratory personnel

#### Training in biosafety procedures

There are four biosafety levels (BSLs), which are combinations of laboratory procedures and methods, safety apparatus, and laboratory facilities, and MPXV is carried out at biosafety level 3 and animal biosafety level (BSL-3/ABSL-3) [68]. High-containment labs (BSL-3/4) must coordinate and communicate globally to share their experiences and lessons learned in order to improve their capacity to respond to threats, as emerging and re-emerging infections and potential bioterrorism acts will continue to pose a challenge to both the medical community and civilian populations worldwide [69].

The following are some developed standards and norms that are required for training scientists and support staff: 1) formal training that consists of three components: (1) classroom-style or didactic theoretical preparation; (2) one-on-one practical training in the facility; (3) mentored on-the-job training; and (4) reevaluation of all personnel working in the BSL-3/4 to ensure that their knowledge and skills remain current [69].

Every researcher who works with experimental animals should be adequately educated to manage the specific species being studied [51]. Also, research study protocol should specifically include the process for handling and caring for experimental animals in a humane manner [70]. Given that animals and humans have comparable levels of sensitivity to pain, suffering, survival instincts, and consciousness, it is the investigator’s duty to keep close tabs on the animals being utilized and spot any indications of discomfort [51].

In the case of exposure, workers should be vaccinated, sick persons detected early and isolated. Also, appropriate hygiene standards are the basic recommendations for treating and preventing monkeypox in occupational settings [71].

Laboratory personnel competency is a crucial component of research because, at the rate at which information is becoming outdated, it can be extremely difficult to maintain proficiency at a suitable level [72]. It’s critical to ensure clinical researchers are qualified since poor clinical research affects public health and takes up investigators’ time and scarce institutional resources [73]. Therefore, continuous professional development (CPD) and lifetime learning are mandatory for registered laboratory professionals because it guarantees that they maintain their competence to deliver safe, legal, and efficient services. Online seminars, virtual laboratory stimulation sessions, conferences, and workshops are a few examples of CPD methods. Because biomedical research needs both theoretical knowledge and proficiency in professional practice and practical methods [74].

A number of frameworks, including the joint task force on clinical trial competency, core competencies in clinical and translational research, and others, have been created to help clinical research professionals and researchers describe the abilities thought necessary for clinical research. However, many of them lack assessment methods or have unvalidated assessment techniques [73].

#### Emergency response and incident management

Strong surveillance and detection systems that allow for the early discovery and prompt control of outbreaks are essential to an efficient MPXV response. At the international level, WHO and its partners coordinate and lead responses using a comprehensive strategy that includes effective access to medical countermeasures and strategic leadership in support of national response operations [23].

Healthcare professionals, those who work with animals and sex workers are at the highest occupational risk for the MPXV [75]. Individuals who have been diagnosed with monkeypox should be quarantined swiftly pending the time the rash scabs come off and such persons should make use of certain household objects that are not to be shared with other family members [1]. Due to the possibility of contaminated-fomite-driven illness, care should be exercised when living with someone who has MPXV. Three weeks after the beginning of symptoms, MPXV DNA has been found on surfaces in homes [76]. The first step in preventing the spread of MPXV from person to person is educating the public about the dangers of MPXV transmission and ways to reduce viral exposure [76].

MPXV has been reported to be readily inactivated by formalin, methanol, and chloroform, although it is resistant to ether and drying. The virus is rendered inactive by heating it to 56 °C for 30 min. Consequently, these substances or techniques might be applied to decontaminate MPXV during exposure incidents [1].

## Challenges and recommendations

### Challenges in implementing safety protocols

Inadequate research infrastructure is one of the most important obstacles to safety during Mpox research [77]. In many developing countries, research facilities are underdeveloped and under resourced, especially in isolated and rural locations where Mpox is very prone to emerge [78]. The effective safety of Mpox research, diagnosis, and management is frequently hampered by inadequate research facilities and restricted laboratory capacity [79]. The risk of laboratory-associated transmission increases in research settings due to the restricted availability of personal protective equipment (PPE) and other essential resources [78]. Due to a lack of sophisticated laboratory facilities, researchers frequently employ general approaches, which can be challenging because mpox shares traits with other febrile viruses, such as chickenpox [80]. The timely execution of control measures, such as contact tracing and patient isolation, is affected by this identification ambiguity as well as the limited availability of essential laboratory supplies and isolation facilities [81]. Because of this, outbreaks have the potential to spread swiftly and overwhelm already vulnerable research systems [81]. The problem is worsened by the lack of qualified personnel to identify and manage mpox [78]. Researchers frequently lack the skills and resources necessary to properly detect and handle mpox, which can result in incorrect diagnoses, ineffective treatment, and increased virus spread [82]. Moreover, the lack of effective procedures to identify, report, and track Mpox cases in real time makes outbreak containment extremely difficult [83].

The inconsistent categorization of mpox research under the proper BSL standards is one of the main obstacles [23]. Because of the aerosol transmissibility and potential for zoonotic transmission, work utilizing MPXV frequently requires BSL-3 or higher facilities in high-income nations [4, 24]. Researchers may, however, carry out comparable work in resource-constrained environments using BSL-2 circumstances, which do not have essential containment techniques like HEPA filtration or negative pressure environments [78, 83].

### Recommendations for best practices

#### Adoption of standardized, evidence-based biosafety protocols

International collaboration is essential for managing mpox outbreaks [83, 84]. To guarantee uniform biosafety procedures, organizations carrying out mpox research should conform to globally accepted standards, such as WHO and Centre for Disease Control (CDC) recommendations [78]. Countries severely affected by mpox should receive financial, technical, and research support from governments, non-governmental organizations (NGOs), and global health organizations [78]. This entails strengthening biosafety systems, expanding laboratory infrastructure, and guaranteeing fair access to established biosafety procedures.

#### Continual assessment and improvement of biosafety measures

Regular evaluations of biosafety methods and infrastructure are crucial to identify weaknesses and resolve developing threats [78]. Investment in research infrastructure is critical to increasing the capacity of research systems to properly respond to mpox outbreaks. For early and precise detection that allows for quick outbreak containment, laboratory capabilities must be strengthened [85]. This entails supplying labs with cutting-edge equipment for Mpox detection and educating research staff on better biosafety protocols [86].

## Conclusions

The challenges with managing animal research safety and outbreaks during mpox research can be linked to several factors involving limitations in facility design and equipment and differences in regional and institutional biosafety standards [78, 87]. Full compliance with established protocols for biosafety, such as the use of suitable containment facilities (ideally BSL-3), the usage of appropriate personal protective equipment (PPE), and stringent cleaning processes, are the key safety practices in animal research on mpox [78]. Training of researchers and laboratory attendants on adherence to established protocols are required to reduce the risks of laboratory transmission [77, 79].

The deficiencies in animal research safety in mpox need to be addressed immediately to keep outbreaks from developing into serious emergencies [83]. Adopting standardized, evidence-based biosafety protocols and the continual assessment and improvement of biosafety measures are the recommended tactics [83]. Underdeveloped countries must build sustainable strategies against the outbreak of mpox in animal research [23, 87]. Interagency collaboration remains super-important in enhancing animal research safety in mpox [78]. Coordination between international and intercontinental agencies such as the World Organization for Animal Health (WOAH), World Health Organization (WHO), and Centre for Disease Control and Prevention (CDC), in collaboration with national and regional institutions, can facilitate the standardization and enhancement of biosafety practices locally, nationally, regionally, and even globally [78]. This can be done by ensuring that the biosafety guidelines are harmonized and facilitating resource-sharing and training programs for researchers [78].

## Data Availability

Data sharing is not applicable to this article as no datasets were generated or analyzed during the current study.

## Abbreviations

ABSL: Animal Biosafety Level
ASPA: Animal Scientific Procedures Act
BSC: Biological Safety Cabinet
BSL: Biosafety Level
CDC: Centre for Disease Control
CIOMS: Council for International organization of Medical Sciences
CNS: Central Nervous System
CPD: Continuous Professional Development
DNA: Deoxyribonucleic Acid
EU: European Union
FSAP: Foundation to Support Animal Protection
HEPA: High Efficiency Particulate Air
IACUC: Institutional Animal Care and Use Commitee
LD50: Median Lethal Dose
MPX: Monkey Pox
MPXV: Monkey Pox Virus
NGO: Non-Governmental Organization
NHPs: Non- Human Primates
NRC: National Research Corporation
PPE: Personal Protective Equipment
RNA: Ribonucleic Acid
SOP: Standard Operating Procedure
UFAW: Universities Federation for Animal Welfare
ULPA: Ultralow Particulate Air
WA: West Africa
WAOH: World Organization for Animal Health
WHO: World Health Organization
